# Improvement of POC-CCA Interpretation by Using Lyophilization of Urine from Patients with *Schistosoma mansoni* Low Worm Burden: Towards an Elimination of Doubts about the Concept of Trace

**DOI:** 10.1371/journal.pntd.0004778

**Published:** 2016-06-21

**Authors:** Paulo Marcos Zech Coelho, Liliane Maria Vidal Siqueira, Rafaella Fortini Queiroz Grenfell, Nathalie Bonatti Franco Almeida, Naftale Katz, Áureo Almeida, Nídia Francisca de Figueiredo Carneiro, Edward Oliveira

**Affiliations:** 1 Schistosomiasis Laboratory, Rene Rachou Research Center, Oswaldo Cruz Foundation (Fiocruz), Belo Horizonte, Minas Gerais, Brazil; 2 Department of Infectious Diseases, College of Veterinary Medicine, University of Georgia, Athens, Georgia, United States of America; 3 Zoonoses Control Centre, Montes Claros, Minas Gerais, Brazil; 4 Clinical Research Laboratory, Rene Rachou Research Center, Oswaldo Cruz Foundation (Fiocruz), Belo Horizonte, Minas Gerais, Brazil; Centers for Disease Control and Prevention, UNITED STATES

## Abstract

**Background:**

Accurate diagnostic techniques for schistosomiasis are essential for prevalence determination and identification of positive patients. A point-of-care test for detecting schistosome circulating cathodic antigen (POC-CCA) has been evaluated for its accuracy in different endemic regions. This reagent strip/dipstick based assay has showed high sensitivity for individuals with high or moderate worm burden, but the interpretation of light infections is less clear, especially for trace readings.

**Methodology/Principal Findings:**

We introduced a urine lyophilization step to the POC-CCA assay to improve its sensitivity and clarify the interpretation of traces. We evaluated POC-CCA sensitivity and specificity within individuals with low parasite burdens in a Brazilian endemic area where a high number of traces were detected. Patients that were positive for other helminths were also evaluated for cross reactions. In all cases, a combined parasitological diagnosis using Kato-Katz (24 slides) and Saline Gradient (1 g of feces) were used as reference. At baseline, diagnosis by POC-CCA (1–2 cassettes) showed 6% sensitivity, inaccurately predicting a low prevalence of *Schistosoma mansoni* infections (2 POC-CCA positives/32 egg positives). After urine lyophilization, the sensitivity was increased significantly (p < 0.05). Prevalence rates changed from 2% to 32% (27 POC-CCA positives/32 egg positives), equivalent to parasitological techniques. Most of the trace readings changed to positive after lyophilization while some negatives turned into traces. Cross reaction analysis confirmed the specificity of POC-CCA.

**Conclusions/Significance:**

Trace readings cannot be primarily defined as positive or negative cases. It is critical to verify case-by-case by concentrating urine 10 fold by lyophilization for the diagnosis. Following lyophilization, persistent trace readings should be read as negatives. No trained technician is needed and cost is restricted to the cost of a lyophilizer and the electricity to run it.

## Author Summary

Schistosomiasis mansoni is a relevant disease affecting millions of individuals in different countries, in particular countries in Africa, and Brazil. Diagnosis performed by Kato-Katz technique for the detection of eggs in stool and a point-of-care test for circulating cathodic antigen detection in urine (POC-CCA) has been evaluated. Both methods have decreased sensitivity when diagnosing patients with low parasite burdens, which can lead to infected individuals not receiving treatment. Here, we focused on interpretation of POC-CCA results in persons with low parasite burdens. We noted a high number (49%) of indeterminate results, including false negatives and trace readings. A urine concentration step was included to improve the test’s sensitivity. Important differences on sensitivity and prevalence rates were noted when comparing diagnosis by POC-CCA before and after urine concentration. Notably, indeterminate results were easily defined after introduction of this step. Cross reaction analysis confirmed the specificity of POC-CCA, with exceptions noted for individuals with hookworm infection. In conclusion, trace readings cannot be primarily defined as positive or negative cases. It is imperative to analyze each case individually by concentrating urine prior to the introduction of treatment, instead of relying on a point-of-care test with indeterminate results.

## Introduction

World Health Organization (WHO) guidelines for control and elimination of schistosomiasis require pre-treatment evaluations of the prevalence of *Schistosoma* infections to inform decisions on how often to treat within endemic areas [1]. The WHO has articulated goals to control the disease by 2020 [2]. Accurate diagnostic techniques are essential for accurate determination of prevalence [3], evaluation of mass drug administration programs [4–8], elimination of the parasite [9–11], and/or drug-resistance and pharmacovigilance [12,13]. To address some of the concerns with the Kato-Katz reference technique, such as the need for evaluation of multiple slides to improve sensitivity, extensive research has been devoted to alternative methods with enhanced sensitivity and specificity for detection of *S*. *mansoni* infections [14].

Sensitivity and specificity of a urine-based point-of-care test (POC-CCA) has been evaluated in different endemic settings to detect schistosome circulating cathodic antigen (CCA) [15–20]. The test’s rapid turnaround and ease of use eliminate the need for multiple sample collections and specialized technicians. In addition, bulk production and purchasing of cassettes, particularly in the context of drug administration programs, have real potential for cost savings [21]. Different studies concluded that reagent strip/dipstick based tests have a good performance in detecting CCA in urine from individuals actively infected with *S*. *mansoni* [22–24]. Results from these studies consistently show higher *S*. *mansoni* prevalence scores by POC-CCA test in comparison to when single, double, quadruple or sextuple Kato-Katz thick smears were used [15, 16, 20, 24]. It was well established that the detection of *S*. *mansoni* increases with the increasing number of Kato-Katz smears examined and this pattern was consistently maintained in epidemiological studies [24–29]. However, controversies are found when discussing POC-CCA sensitivity in low endemicity sites, showing a consistent performance only in patients with moderate or high parasite burden [15, 16, 20–23]. It is unclear whether persons with positive POC-CCA readings who are Kato-Katz negative are truly infected or if they have false positive POC-CCA results.

Although several analyses have been performed to explore POC-CCA performance [30], no data have been published concerning cross reaction of the test with helminths or other parasites. In addition, as a qualitative method based on an individual interpretation, doubts have been raised on how to differentiate the “trace” readings between low infection, cross reaction or even no active infection. Instead of being consistent about the meaning of the trace result, authors have chosen to perform a two-way analysis and consider traces as sometimes positive and sometimes negative. This produces vast discrepancies in prevalence intensities [15–18, 20–24, 30]. Thus in this paper we try to clarify the implications of a trace result. A better understanding of the interpretation of trace results is imperative for POC-CCA application in schistosomiasis control programs worldwide, especially in low endemicity areas that need particularly accurate diagnoses. Otherwise, praziquantel may be given to healthy individuals in error if a person has been incorrectly diagnosed.

Adjustments to the assay’s implementation or interpretation of its ability to diagnose individuals with light infections are still needed. We introduced a single step in an attempt to improve the sensitivity and interpretation of POC-CCA trace result. Moreover, we show data from a Brazilian endemic region using the POC-CCA as a diagnostic tool. This improvement was then evaluated within individuals from a low endemicity area where a number of trace results were noted. The work includes initial diagnosis of patients with low parasite leads, that are commonly noted in endemic settings, and a comparison with a combined reference of 24 Kato-Katz slides plus 2 analyses (1 g of feces) by the Saline Gradient technique. The potential implications are discussed.

## Methods

### Ethics statement

This study was approved by the Ethical Research Committee of the Rene Rachou Research Center (CEPSH/CPqRR 03/2008) for human studies. All participants received an explanation of the study objectives. In addition, written informed consent was obtained before admission to the project. Parents/guardians provided written consent on behalf of all child participants. After parents/guardians had signed the informed consent, children received an explanation about the procedure, in a clearly explained language, and had the right to express their opinion. Procedures were performed in the presence of parents/guardians. Samples were coded and results were treated confidentially. Participants that were positive for parasitological tests were clinically examined by a physician and treated with praziquantel (60 mg/Kg for children and 40 mg/kg for adults) and albendazole (400 mg), in single oral dose, as recommended by the Brazilian Health Ministry.

### Community survey and sample collection

This study was conducted in Estreito de Miralta, a schistosomiasis-endemic region, next to the city of Montes Claros, Minas Gerais, southeastern Brazil, approximately 500 km from the state capital (Belo Horizonte). This endemic setting has a population of 163 individuals that had not received treatment for schistosomiasis within the last 2 years and had a low migration index. A schistosomiasis prevalence of 10.34% had been previously reported by the Montes Claros Zoonosis Control Centre in 2008. Positive patients were treated with praziquantel. The present study was conducted in 2013. Positive individuals were identified and treated, as recommended by the Brazilian Ministry of Health. Those patients submitted new fecal samples 30 days post-treatment for parasitological diagnosis and were retreated if needed. All the 84 individuals that provided urine samples (46 females and 38 males, 1–86 years old) were included in this study [31].

### Stool samples

One sample of stool per individual of all the 163 residents was provided for Kato-Katz thick smear examination [31], performed with a total of 24 slides, a total of 1 g of feces examined per individual (24 x 41.7 mg of feces). Results were expressed as eggs per gram (epg) of feces, calculated by the number of *S*. *mansoni* eggs on the 24 slides.

Fecal samples were also analyzed by Saline Gradient test (with two portions of 500 mg, total of 1 g of feces), as previously described [32]. Briefly, the separating column holding a filter was pre-wet with 3% saline solution. The separating column was filled with a fecal suspension prepared by diluting 500 mg stool sample in 3 ml of 0.9% saline solution. The saline flow was adjusted to 10 drops/min. After the slow and continuous flow of the 3% saline solution, low-density fractions were discharged and sediment was retained on the bottom of the latter column. Eggs, which had high density remained on the surface. The fractions containing eggs was moved to glass slides and examined under a bright field microscope. In order to detect other helminths, both parasitological methods were used as diagnostic tools. Results are expressed as epg.

### Urine samples

Each participant was asked to provide one midstream urine sample. Urine samples of the 84 individuals who provided urine were lyophilized to concentrate antigens. Briefly, the urine samples were aliquoted into vials (5 ml/vial), frozen at -20°C for 2 h and then overnight at -70°C and subjected to freeze-drying in a lyophilizer (Alpha 2–4 LD plus, Martin Christ Gefriertrocknungsanlagen GmbH, Osterode am Harz, GE) at 0.023 mbar and -55°C for 24 hours. Lyophilized samples were resuspended with water in a final volume of 0.5 ml, resulting in 10 times concentrated urine samples.

### POC-CCA testing

POC-CCA tests were performed in accordance to the manufacturer’s instructions (Rapid Medical Diagnostics), before and after lyophilization. When a trace reading was obtained, a second cassette was used for confirmation. Briefly, one drop of urine was placed in the cassette’s well. Once it was absorbed, a drop of the kit buffer was placed in to the same well. Results were read after 20 min of test development. The tests were read as invalid when the control band did not appear or when the tests were left to develop for more than 25 min. Results were scored as “0” if the result was negative (i.e., the control line developed, but no test line appeared); trace if a very light test line appeared, “1+” if a test line appeared, but its color was less intense than that of the control line; “2+” if the test and control lines were equally intense in color; and “3+” if the test line’s color had a higher intensity than the control line’s color.

### Data analyses

Data collected from the evaluations were entered into an Excel data base and analyzed by Minitab statistical software (Minitab Inc, United States of America). The reference was defined as any positive slide performed for each individual stool sample by Kato-Katz or Saline Gradient technique. The sensitivity and specificity were determined with OpenEpi software (OpenEpi, Brazil) [33]. The agreement between the parasitological methods and POC-CCA were assessed by Kappa (k) statistics calculated by GraphPad (GraphPad Software, Inc., USA): k < 0.01 no agreement; k = 0.01–0.20 ‘poor’; k = 0.20–0.40 ‘fair’; k = 0.40–0.60 ‘moderate’; k = 0.60–0.80 ‘substantial’; k = 0.80–1.00 ‘almost perfect’ [34].

## Results

### Prevalence estimates

**Fig 1:**
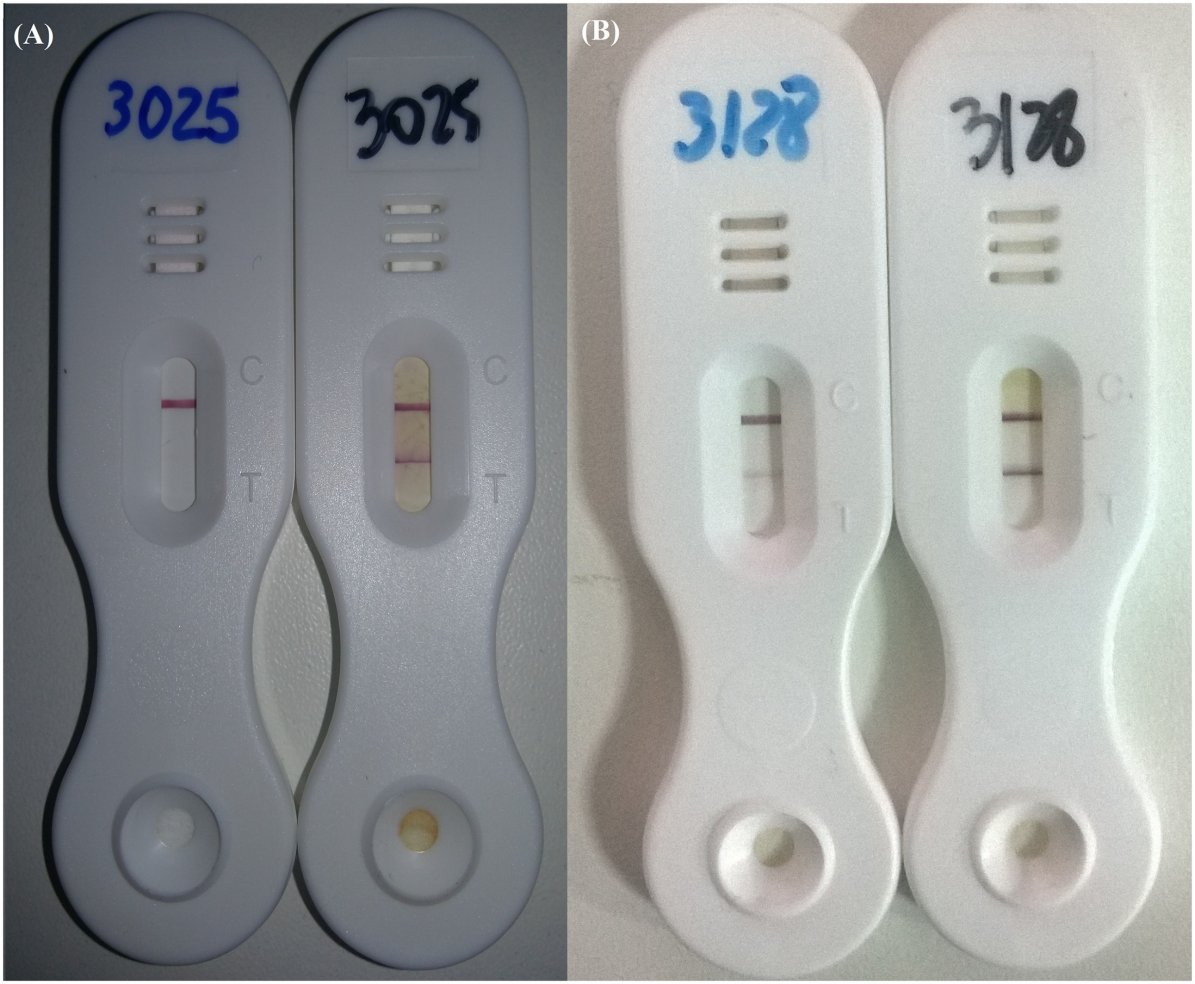
POC-CCA result before and after lyophilization step for 10 times concentration of urine. (A) Diagnosis of the same individual with negative result before urine lyophilization and positive result after urine lyophilization. (B) Diagnosis of the same individual with trace result before urine lyophilization and positive result after urine lyophilization.

A total of 84 individuals participated in this study providing stool and urine samples. Using the combined results of Kato-Katz and Saline Gradient (both 1 g of feces/individual), no egg was detected in 52 individuals. Within those negative cases, POC-CCA was also negative for 42 individuals and 10 egg negative individuals had trace results. Together, 18 individuals presented eggs in stool for either parasitological test (7 by Kato-Katz, 7 by Saline Gradient, and 4 by both methods). Within those 18 individuals, based on POC-CCA test, 3 were negative (2 to 38 epg), 13 presented trace (1 to 55 epg) and only 2 were positive (8 and 16 epg). When two cassettes were used, results were reproduced for 75% of the urines. When cassette performances varied, one result was negative and the other was a trace for the same urine sample, but never a positive result. Table 1 shows the individual descriptive data for the three diagnostic tests. It is important to note that from the 49 individuals that were negative for POC-CCA, 3 presented eggs in only 2 Kato-Katz slides. The estimated prevalence in Estreito de Miralta by each of the three tests is shown in Table 2. For POC-CCA, analysis was first done by the direct application of the urine sample on the cassette and a second analysis was performed after 10 fold concentration by lyophilization. Kato-Katz and Saline Gradient had the same prevalence prediction of 30%. This prevalence rate was much higher than the one predicted by POC-CCA (1–2 cassettes) using unconcentrated urine of 2%. This 2% rate turned into a prevalence of 32% after the urine samples were concentrated, achieving a similar estimated prevalence as either parasitological technique. Fig 1 shows how readings obtained for the same individuals before and after urine lyophilization from negative to positive and from trace to positive, respectively.

**Table 1:** Schistosomiasis diagnosis data of the individuals from Estreito de Miralta, Brazil, based on the POC-CCA results and epg obtained by parasitological methods.

POC-CCA	Kato-Katz		Saline Gradient
(1–2 cassettes)	(2 slides)	(24 slides, 1 g of feces)	(1 g of feces)
49 negative			
0 epg	46	42	43
≤10 epg	3	5	4
11–30 epg	0	0	1
31–60 epg	0	2	1
61–80 epg	0	0	0
33 trace			
0 epg	24	17	16
≤10 epg	9	12	15
11–30 epg	0	1	1
31–60 epg	0	2	1
61–80 epg	0	1	0
2 positive			
0 epg	0	0	0
≤10 epg	2	1	1
11–30 epg	0	0	1
31–60 epg	0	1	0
61–80 epg	0	0	0
Total			
84	84	84	84

**Table 2:** Schistosomiasis positivity based on POC-CCA, Kato-Katz and Saline Gradient among 84 individuals, Brazil.

	Prevalence (%)
POC-CCA	
Before lyophilization	2
After lyophilization	32
Kato-Katz	30
Saline Gradient	30

### Sensitivity, specificity and POC-CCA performance

The sensitivity and specificity of the POC-CCA before and after lyophilization of urine samples were estimated with 95% exact CIs and are shown in Table 3. Respectively before and after lyophilization, POC-CCA presented 6% and 56% sensitivity and, 100% and 83% specificity. The concentration step improved POC-CCA performance, especially when trace results represented negative cases (Tables 4 and 5). Before lyophilization, 49 individuals were detected as negative, 33 presented trace and only 2 were positive for *S*. *mansoni* infection. Then, 13 initially negative individuals turned into traces and 11 into positives, after lyophilization. Of the 13 new traces, 10 individuals had no *S*. *mansoni* eggs in their stool, but 3 individuals were egg positive with 3, 8 and 52 epg of feces. By contrast, 4 individuals of the 11 concentrated urine positives presented 1, 2, 3 and 55 epg and the POC-CCA reading intensities were 1+, 1+, 3+ and 3+, respectively. However, 7 of the post-concentration positives were from egg negative patients; 5 of them had 1+ results, and one each had 2+ and 3+ results. Supporting initial data that considered trace as negative for infection, 15 traces turned into positives after lyophilization. Among those, 13 cases presented eggs in stool (3 patients with 1 epg, 1 with 2 epg, 1 with 7 epg, 1 with 8 epg, 2 with 9 epg, 2 with 10 epg, 1 with 15 epg, 1 with 38 epg and 1 with 55 epg). The two exceptions that had no *S*. *mansoni* eggs were positive for hookworms eggs.

**Table 3:** Sensitivity and specificity obtained by POC-CCA evaluated against a combined reference techniques of 24 Kato-Katz slides and Saline Gradient (1 g of feces) before and after lyophilization.

	Sensitivity (%), 95% CI	Specificity (%), 95% CI
Before lyophilization	6	100
After lyophilization	56	83

**Table 4:** POC-CCA performance on schistosomiasis diagnosis before and after lyophilization against parasitological methods.

	POC-CCA (1–2 cassettes)	POC-CCA after lyophilisation (1–2 cassettes)	Kato-Katz/Saline Gradient
Negative	49	32	52
Positive	2	27	32
Trace	33	25	-
Total	84	84	84

**Table 5:** Descriptive changes on the POC-CCA results before and after lyophilization process and comparison against parasitological results.

	N	Description
Negatives to traces	13	10 patients had no eggs in stool, although 1 presented hookworms eggs. The other 3 patients had 3, 8 and 52 epg of feces
Negatives to positives	11	4 patients with 1, 2, 3 and 55 epg and 7 individuals with no eggs in stool
Traces to positives	15	13 patients showed 1–55 epg while 2 individuals had no eggs in stool, but both presented hookworms eggs

The Kappa index was used to compare parasitological data and POC-CCA urine assay and to better understand the implications of trace results. ‘Poor’ agreement was obtained when comparing parasitological and POC-CCA assays on unconcentrated urine (0.002 and 0.076, respectively for trace as positive and negative). The agreement changed to ‘moderate’, with a Kappa Index of 0.401, after urine samples were concentrated, but only when considering trace as negative for schistosomiasis. No change in the agreement between parasitological and POC-CCA data was noted when considering trace as positive for concentrated urine (0.125).

### Cross reactivity evaluation

Some individuals presented no *S*. *mansoni* eggs in stool, even after an extensive search, but had eggs of other helminths including hookworms, *Hymenolepis nana*, *Enterobius vermicularis* and *Ascaris lumbricoides*. No study participants were co-infected. Because it is common to find individuals with helminth infections other than schistosomiasis in endemic areas, we evaluated the POC-CCA on those individuals to determine whether infection with these other worms is associated with a false positive result. As shown in Table 6, among 7 individuals who were negative for schistosomiasis but positive for hookworms, 3 had a negative POC-CCA result and 4 had a trace result. Three *S*. *mansoni*-negative individuals were positive for *H*. *nana*. One was negative by POC-CCA, and 2 had trace reactions. All 4 individuals who were negative for schistosomiasis but positive for *E*. *vermicularis* and the one who was positive for *A*. *lumbricoides* had trace results. In order to see if traces would turn into positives after urine concentration, we evaluated the lyophilized urine samples for POC-CCA. In 12 of 15 patients, the POC-CCA results were the same before and after lyophilization. Three exceptions were found, one POC-CCA negative changed to trace for a person with hookworms infection, one pre-concentration trace result changed to 1+ for a patient with hookworms and pre-concentration trace result changed to a negative result for a person with *E*. *vermicularis* infection.

**Table 6:** Cross reactivity analysis of duplicate POC-CCA before and after lyophilization of urine samples detected by the combined reference parasitological diagnosis for individuals negative for schistosome eggs.

	Results POC-CCA	
	Before lyophilization	After lyophilization
Hookworms		
Individual 1	Negative	Negative
Individual 2	Negative	Negative
Individual 3	Trace	Trace
Individual 4	Trace	Trace
Individual 5	Trace	Trace
Individual 6	Negative	Trace
Individual 7	Trace	Positive (1+)
*Hymenolepis nana*		
Individual 1	Negative	Negative
Individual 2	Trace	Trace
Individual 3	Trace	Trace
*Enterobius vermicularis*		
Individual 1	Trace	Trace
Individual 2	Trace	Trace
Individual 3	Trace	Trace
Individual 4	Trace	Negative
*Ascaris lumbricoides*		
Individual 1	Trace	Trace

## Discussion

The POC-CCA test is a promising technique that uses a nitrocellulose strip coated with monoclonal antibody to detect schistosome CCA antigen in urine samples. The antigen binds to the labelled monoclonal antibody immobilized on the nitrocellulose strip when urine from infected individuals flows through the strip. A band becomes visible with the binding of labelled monoclonal antibody [35]. Thus far, stool microscopy is the recommended diagnostic ‘gold’ standard, but individuals with low parasite burdens (i.e. < 100 epg of stool) are often missed [3, 6, 14–18, 21, 22, 25–29] and is indispensable to have technical expertise in microscopic recognition of intestinal parasite eggs. In the last few years, the POC-CCA has been extensively tested in areas endemic for schistosomiasis on the African continent [15–24, 30, 35]. These studies have shown the efficiency of a single or a double POC-CCA analyses in comparison to one or two Kato-Katz thick smears as the diagnostic standard. In all the cases, same relation was seen—for high parasite burden, higher positivity of POC-CCA is obtained.

Although an increasing number of studies evaluating POC-CCA has been reported in endemic areas of Africa, there is so far no study evaluating its performance in Brazilian affected areas. Jointly, only 52 countries of the 78 countries considered endemic for schistosomiasis have populations requiring preventive chemotherapy, according to WHO [36]. It is a consensus that endemic areas in Africa and Brazil have different profiles regarding prevalence and morbidity. In this regard, we have assessed the accuracy of POC-CCA in a Brazilian endemic area where the parasite burden is low (1–80 epg). Our data showed that a number of trace readings was obtained (33/84 individuals) among whom 17 were negative and 16 were positive using parasitological techniques. Correspondingly, the sensitivity of the POC-CCA test used to evaluate prevalence was poor, as the prevalence rate was estimated as 2% when 1–2 cassette tests were performed. Kato-Katz is often criticized for its declining sensitivity when egg count intensities decrease, but the same situation was noted with POC-CCA under these conditions. POC-CCA seems to be appropriate for the diagnosis of *S*. *mansoni* when the prevalence is above 25% and no recent control efforts have been implemented [16]. Our findings show that patients from areas of low endemicity are difficult to detect. So, we propose to add a urine concentration step to the POC-CCA methodology to improve its sensitivity in low endemicity areas of Brazil. With this new step, the prevalence rate changed from 2% to 32%, achieving a comparable rate of Kato-Katz and Saline Gradient, performed on 1 g of feces (30% on both cases).

The efficient identification of infected populations warrants effective chemotherapy, allows the development of new efforts toward elimination, including control interventions, assessment of drug efficacy, and patient management [4, 16, 37–38]. We must emphasize the importance of an accurate diagnosis at the individual and population level. By considering traces as positive, treatment may be performed inappropriately. On the other hand, infected patients could be deprived of receiving praziquantel treatment when traces are considered negative.

Treatment-based control programs worldwide have been successful in reducing infection intensity and the number of persons with severe schistosomiasis. Conversely, transmission remains active in several endemic areas, and subtle but persistent morbidities are often found in persons with low-level reinfections, this is routinely seen, in Brazil. Accurate case-finding is indispensable for the effective execution of control programs [39]. Each trace reading must be individually analyzed since it may report a positive (low epg) or a negative situation, or even a cross reaction case. For urine tests that are scored as trace, we propose a lyophilization step of the urine to concentrate the sample. The introduction of this step in the POC methodology showed a clear diagnostic result. We show here that after lyophilization of urine, all remaining traces were negative. In addition, 15 traces turned into positive cases, 13 of which had a very low number of eggs in stool (1 to 55 epg) and the two others, although negative for *S*. *mansoni* eggs, were positive for hookworms. The Kappa index changed from ‘poor’ to ‘moderate’ agreement when considering concentrated trace results as negative when compared to parasitological data.

When analyzing sensitivity, was 6% when we followed the manufacturer’s instructions. After the lyophilization, sensitivity increased to 56%. Considering the different profile of Brazilian endemic areas, it is relevant to compare the sensitivity rates achieved by Kato-Katz parasitological assay with the one achieved by POC-CCA. It is noticeable that increasing positive rates are obtained as slides are augmented in number, moving from 38.9, 43.5, 49.1, 50.0, 52.8 and 53.7, respectively for 1, 2, 3, 4, 5 and 6 slides [25]. Definitely, 6% is an unacceptable sensitivity rate for a reference diagnostic method, but a rate of 56% is comparable to the performance of several Kato-Katz slides after the concentration of urine.

Differential diagnosis is also important since co-infection between helminths is commonly seen. In those cases, treatment may require different drugs. Only parasitological assays are capable of revealing differential egg identification, but the search for eggs in stool needs a trained and experienced technician. We tested POC-CCA cross reaction by analyzing urine samples of positive patients for other helminths (hookworms, *H*. *nana*, *E*. *vermicularis*, *A*. *lumbricoides*). Trace readings were seen for the POC-CCA assay (in individuals positive for hookworms, *H*. *nana*, *E*. *vermicularis* and *A*. *lumbricoides* eggs). If these traces were considered positives, as most of the authors do in Africa, we would had 73% of individuals incorrectly receiving praziquantel, instead of the correct drug (Albendazol), which would result in untreated patients with persistent infection and morbidity.

The POC-CCA test is particularly well-suited to accurately demonstrate moderate to heavy *S*. *mansoni* infections and can be considered as a useful method for diagnosis in peripheral health centers and schistosomiasis control programs [16], but it does not present accurate results for low infections, as presented here unless the lyophilization step is included. A new potential diagnostic method called UCP-LF CAA has been tested for its accuracy as a urine-based up-converting phosphor-lateral flow circulating anodic antigen assay. The UCP-LF CAA assay showed high sensitivity for the diagnosis of *S*. *haematobium* in low-endemicity settings. According to the authors, the availability of scanners to analyze the UCP-LF CAA strip is a major step toward POC applications in poor resourced sites to accurately identify low (30 pg CAA/ml serum; equivalent to about 10 worm pairs) to heavy *Schistosoma* infections [40, 41].

The improvement of diagnostic methods with high sensitivity and specificity, and of simple execution and low cost will be vital for the accomplishment of the goals recently established by WHO [2]. These goals address the transmission control of schistosomiasis worldwide. Except for Africa, the transmission interruption should be accomplished by the end of 2020. For countries in the African continent, this goal should be achieved by 2025. The improvement of the POC-CCA test described in this study reinforces the possibility of introducing this methodology within the WHO schistosomiasis control proposal [2], not for all populations due to the obvious logistical difficulties, but as a tool to obtain additional data would that allow accurate interpretation of POC-CCA results in areas of low prevalence.
